# Books Review

**Published:** 1869-10

**Authors:** 


### Books Review.

*Transactions of the Medical Society of the State of Pennsylvania, at its Twentieth Annual Session, held at Erie, June*, 1869.

Of the proceedings of the Society, the most important items are the resolutions of Prof. Gross upon medical training of nurses. and resolutions of a committee of the American Medical Association upon medical education, one of which reads as follows :

" *Resolved*. That this association earnestly requests each State Medical Society to appoint annually, one or more Boards of Examiners, composed of five thoroughly qualified members, whose duty it shall be to meet at suitable times and places, for the examination of all persons, whether graduates or not, who propose to enter upon the practice of medicine in their respective States, except such as have been previously examined and licensed by a similar board in some other State."

Other resolutions follow, going to establish independent State Boards of examiners, and ignoring the diploma as evidence of a medical education.

This matter of "resolutions" upon the subject of medical education is about "played out." It reminds us of the showmans Tiger—"The Royal Tiger of Bengal—born in the wilds of Arabia and fed on the milk of a goat," which repeated for the ten thousandth time becomes tiresome to every one who makes long visits to his Royal Tigership. However, some good may come of resolutions, provided none of them are ever put in practice.

Educating nurses is, also, all very well, provided when educated a very little, they do not resolve themselves into a society of female practitioners who would not understand nursing. but would know all the "profound mysteries of the healing art." We are in favor of educated nurses, but we think in the present reign of equal-rights, nothing could be depended upon from them in the way of nursing, but everything might be expected as practitioners of medicine and surgery.

This volume contains an interesting and instructive address from the President, John Corwin, M. D., also a valuable report on intemperance as a disease.

JOSEPH PARRISH, WM. B. ATKINSON,
EDWARD WALLACE, JAMES KING,
JACOB PRICE.
Committee.

Papers on admission of patients into Insane Asylums; on Inspection of Drugs; Rupture of the Uterus; Use of Stimulants by the profession, and description of a new instrument for treatment of Lateral Curvature, together with reports of County Societies, comprise the volume. Its papers are highly creditable to the authors and to the Society, and we regret that space does not permit more extended notice.

---

*Meyer's Medical Electricity.* Translated from the German. By WILLIAM A. HAMMOND, M. D. D. APPLETON & Co., New York, 1869.

In examining this book, with the view of giving our readers some idea of its scope and value, we have turned over unread, the first chapters of the work, and propose only, at present, to examine what the author says of electricity as a curative agent, since this is the subject of first importance to practicing physicians.

He says, in substance, that electricity has been employed with success in medicine, surgery and obstetrics.

The diseases in which electricity has shown itself most efficacious are those that attack the nerves, and those that depend upon anomalous secretions and excretions.

"In the chirurgical art it has achieved results not less satisfactory, and has won an important and permanent place, not only in consequence of the application of electro-thermic processes in galvano-caustic operations, and of electro-chemical processes in the cure of varices and aneurisms; but also in the influence of the electric current, as scientifically established and practically confirmed, in the dispersion of exudations and tumors." The Editor remarks, in this connection, "a point of great interest, and diagnostic importance, is the use of the electric current in discovering the locality of a metalic body which has been forcibly projected into the system," and, in illustration, relates how Nelaton determined that the rifle-ball was still in Garabaldi's ankle. The author mentions several operations theoretically possible which have been experimentally proven, such as the solution of calculi in the bladder, and the removal of poisonous metals from the system, but have been practically utilized in but few cases.

In obstetrics, finally, the electric current has been applied, by the English, to excite the activity of the parturient efforts, and to arrest metrorrhagia, and more recently by the French to overcome version and prolapsus of the womb.

The treatment of neuralgia is mainly with the electric pencil, the physiological action of which, he says, is probably the same as in all epispastica, namely, reflex, which, however, has the advantage over the other epispastic preparations in common use, in the suddenness of the intense pain it excites, and the facility with which the pain may be renewed. Many cases are published in detail, showing the effects of electricity variously applied, in neuralgic affections, which, when grouped and considered, leave upon our minds no very high opinion of the efficacy of this agent, always ourselves disgusted with moxa, however applied.

Cases of Anæsthesia, deafness, impotency, spasms, paralysis, locomotor ataxia, incontinence of urine, lead paralysis, etc., etc., are related in which satisfactory results were obtained in remarkable cases of articular and rheumatic exudations. Suppressed secretions and excretions are reported cured by electricity, under the observation of the author. Space prevents our giving further detail of the cases presented or the claims instituted for electricity as a curative agent. Upon one other subject, however, we must remark a word. Concerning the dispersion of tumors, the author says: " Attempts were some time ago made to remove, with the electric current, infiltrations in lymphatic glands, goitre, ganglia, and similar swellings," and then proceeds to mention the physicians and describe the growths removed, and closes by saying: " A. Becquerel, however, and others, maintain that electricity has no influence whatever on glandular swellings."

In reply he says: " I am able to oppose this opinion decidedly. I will now report two cases, in the first of which I dispersed an infiltration of lymphatic glands of the size of a hens egg, while in the second case I reduced to a minimum the greatest tumor probably ever treated with electricity." He applied to a tumor of stony hardness in the neck, the size of a childs head, electricity for several years, making two hundred and seventy-three applications in all, and when the treatment was finished, the transverse diameter of both sides of the neck differed hardly two inches.

We have examined the work mainly to ascertain what practical suggestions it contains, what the author really claims for electricity as a curative agent. He claims considerable, and hopes for yet greater results. We have mainly held the same opinion of electricity expressed summarily by A. Becquerel, substituting disease in place of glandular swellings, but perhaps this is too sweeping, for it appears from the author, and by many others, a powerful agent, rightly and perseveringly applied. This author is worthy of careful study, for certainly he has presented the whole subject in unexceptionable manner. The translator, also, has done a real service to the English profession, both in thus presenting to them the views of the German author, and by notes and additions, greatly increasing the value of the work.

---

*A Treatise on the Diseases and Surgery of the Mouth, Jaws and associate parts.* By JAMES E. GARRETSON, M. D., D. D. S., with steel plates and numerous wood cuts. J. B. LIPPINCOTT & Co., 1869.

Our author gives first, the Anatomy of the mouth and face, and introduces several well executed wood cuts which illustrate the appearances and the relation of parts. He then describes Dentition and its associate lesions, though this last he does not regard as coming strictly within the scope of a work on surgery. Upon this subject he says : "In the first place we have to remark, that the process of dentition, while a physiological one, is yet like that of utero-gestation, one of continuous irritation. Of the meaning of this word irritation, every surgeon and every physician has, in his mind, quite enough reminiscences. Irritation, then, is the matter of consideration in all and every of these associative lesions, if, happily, in such cases, we could exactly appreciate and exactly control such irritations, we should of course abort or resolve the results. It is not. however, by any means to be estimated that all infantile diseases are influenced by, or indeed even remotely associated with dentition. Mistakes of this nature are quite too frequently made, and infants are tortured, and in many instances have the existing disease, aggravated, by the lancing and cutting which follows." After a very sensible and well considered article upon this subject, he proceeds to describe the "Anomalies of second dentition and their Surgical Relations," and passes to the teeth, their diseases and the means of cure. He also introduces chapters upon Anasthesia, general and local, the glands, gums, caries, necrosis, tumors of the mouth diseases of the Antrum of Highmore. Neuralgia, wounds of the mouth, Ozena, fractures and dislocations of the maxilla, operations upon the lips and cheek, aphthae, diseases of the tongue, palatine defects and their treatment, both surgically and with obturators, and lastly, exsection of the maxillary bones. The author does both himself and his subjects most ample justice, and the work must prove very valuable to both the dental and medical professions ; practitioners of both will be instructed and greatly assisted by its teachings.

---

*The Science and Art of Surgery.* By JOHN ERICHSEN. From the fifth enlarged and carefully revised London edition, with additions by JOHN ASHURST, M. D. Philadelphia: HENRY C. LEA, 1869.

This edition of Erichsen's Surgery comes to us in so much enlarged and improved form that we did not at first recognize it as Erichsen's Surgery; however, upon careful inspection, we find a revision and enlargement of the old and justly popular work. It embodies the surgery of the present day as well as the teachings of former times, and is so comprehensvie in its scope that it may be said to be a complete practical guide for students and practitioners.

This edition of the Erichsen's surgery is also made vastly more valuable than former ones, by the addition of illustrative cuts, nearly seven hundred of which are scattered through the work, thus rendering the text more forcible and much more easily understood. These representations are so perfect that they are only to be seen to more fully impress upon the mind the nature and appearances of many diseased conditions than could possibly be done by pages of written description. The work contains near thirteen hundred closely printed pages, thus affording a complete description of nearly every important surgical affection. It is also eminently a practical one, and evidently designed to afford the practitioner a safe and reliable guide, and the student of surgery a comprehensive and complete text-book. The science and art of surgery are combined in proper proportions to meet the wants of the student, as well as the necessities of the busy physician.

Correct diagnosis in surgery is of the very first importance; and the author of this book appears to be fully alive to this fact, and throughout the whole work has not only given the symptoms of each affection, but also the symptoms and appearances of other diseases with which it is liable to be confounded, as well as all the means by which they may be known from each other. Perhaps, in this respect, it is unequaled by any book on surgery of its scope and design. Certainly in this, as in other respects, it is very complete; and, we have no doubt, will continue to be a great favorite with both medical students and practitioners.

---

*Treatment of Lachrymal Affections.* By Professor ARLT of Vienna. Translated by JOHN F. WEIGHTMAN, M. D. LINDSAY & BLAKISTON, Philadelphia, 1869.

It appears that Professor ARLT, noticing at the Ophthalmological Congress held in Paris, the various views prevalent as to the treatment of Lachrymal Fistula, has written a pamphlet upon the subject, and as illustrating the subject has introduced cuts which assist greatly in understanding the text. The translator says : " By following the rules laid down by him, the lachrymal probe may nearly always be introdued with ease and safety, where previously the introduction was attended with much difficulty."

---

*Lindsay & Blakiston's Physician's Visiting List for* 1870. Philadelphia.

We have to acknowledge the receipt of LINDSAY & BLAKISTON'S Visiting List for 1870, a pocket companion now regarded as indispensable by nearly all physicians, especially those practicing in the cities and larger villages. It has the usual table of contents, now too well known to require mention, and appears in its usual excellent tuck binding and tasty finish. It is for sale by all booksellers, and may generally be obtained of, or through druggists.

